# Evidence for Training-Induced Changes in miRNA Levels in the Skeletal Muscle of Patients With Type 2 Diabetes Mellitus

**DOI:** 10.3389/fphys.2020.599651

**Published:** 2020-12-03

**Authors:** Sarah Simaitis, Benedikt Schulte-Körne, Thorsten Schiffer, Wilhelm Bloch, Hans-Georg Predel, Klara Brixius, Christian Brinkmann

**Affiliations:** ^1^Department of Molecular and Cellular Sport Medicine, Institute of Cardiovascular Research and Sport Medicine, German Sport University Cologne, Cologne, Germany; ^2^Department of Preventive and Rehabilitative Sport Medicine, Institute of Cardiovascular Research and Sport Medicine, German Sport University Cologne, Cologne, Germany; ^3^Outpatient Clinic for Sports Traumatology and Public Health Consultation, German Sport University Cologne, Cologne, Germany; ^4^IST University of Applied Sciences, Düsseldorf, Germany

**Keywords:** diabetes, miRNA - microRNA, epigenetics, glycemic control (HbA1c), training, exercise

## Abstract

Physical training can improve glycemic control in patients with type 2 diabetes mellitus (T2DM). However, the underlying mechanisms are not entirely clear. An interesting piece of the puzzle could be the regulation of micro-RNAs (miRNAs). They are important modulators of protein expression. Some miRNAs were found to be both linked to poor glycemic control/insulin resistance (with evidence from *in vivo* and/or *in vitro* studies) and dysregulated in the skeletal muscle of T2DM patients. This pilot study examines whether a 3-month endurance training program [three times a week, 70–80% peak heart rate (HR_peak_)] can down-regulate their levels in T2DM men (*n* = 7). One skeletal muscle biopsy sample was obtained from each patient at T1 (6 weeks pre-intervention), one at T2 (1 week pre-intervention) and one at T3 (3–4 days post-intervention). miRNA-27a-3p, −29a-3p, −29b-3p, −29c-3p, −106b-5p, −135a-5p, −143-3p, −144-3p, −194-5p, and − 206 levels were determined by RT-qPCR. Friedman ANOVA and post-hoc tests showed that miRNA-29b-3p, −29c-3p and -135a-5p levels were significantly reduced post-training (T3 vs. T2 and/or T1). Glycated hemoglobin (HbA1c) and HOMA insulin resistance index did not change significantly. However, HbA1c was reduced in 6 of 7 patients post-training. Furthermore, Spearman’s rank correlation analyses with all values from all time points showed significant negative associations between miRNA-29c-3p, −106b-5p, −144-3p and −194-5p levels and cardiorespiratory fitness (VO_2peak_). The study results imply that regular exercise and improving one’s physical fitness is helpful for the regulation of skeletal muscle miRNAs in T2DM patients. Whether or not changes in the miRNA profile can affect the clinical situation of T2DM patients warrants further research.

## Introduction

Regular physical activity is a crucial element in the therapy of patients with type 2 diabetes mellitus (T2DM), and it has been demonstrated that physical training can improve glycemic control in most of the patients (Chudyk and Petrella, 2011). However, the underlying molecular mechanisms of its health-related effects have not yet been clarified in all details. The regulation of micro-RNAs (miRNAs) could play an important role in this context. miRNAs are important modulators of protein expression and involved in organ cross-talks as it has been shown that they can be transported in blood from one organ to another where they can post-transcriptionally regulate cellular processes by base pairing with messenger RNAs (Yu et al., 2016). Some miRNAs have been linked to insulin resistance *in vivo* and/or in cell models *in vitro* (Chakraborty et al., 2014; Dahlmans et al., 2016, 2017; Jones et al., 2017). To date, the influence of regular physical training on these miRNAs in the skeletal muscle of T2DM patients has not been examined. The present study thus aims to determine whether physical training (endurance type) can change levels of miRNAs -27a-3p, −29a-3p, −29b-3p, −29c-3p, −106b-5p, −135a-5p, −143-3p, −144-3p, −194-5p, and −206, which were all found to be negatively linked to variables of glycemic control and which can be dysregulated in the skeletal muscle of patients with T2DM (Gallagher et al., 2010; Jordan et al., 2011; Karolina et al., 2011; Zhang et al., 2013, 2017; Latouche et al., 2016; Zhou et al., 2016; Dahlmans et al., 2017). The hypothesis that regular physical endurance exercise down-regulates the afore-mentioned miRNAs in the skeletal muscle of T2DM patients was tested.

## Materials and Methods

### Study Design

**Figure 1:**
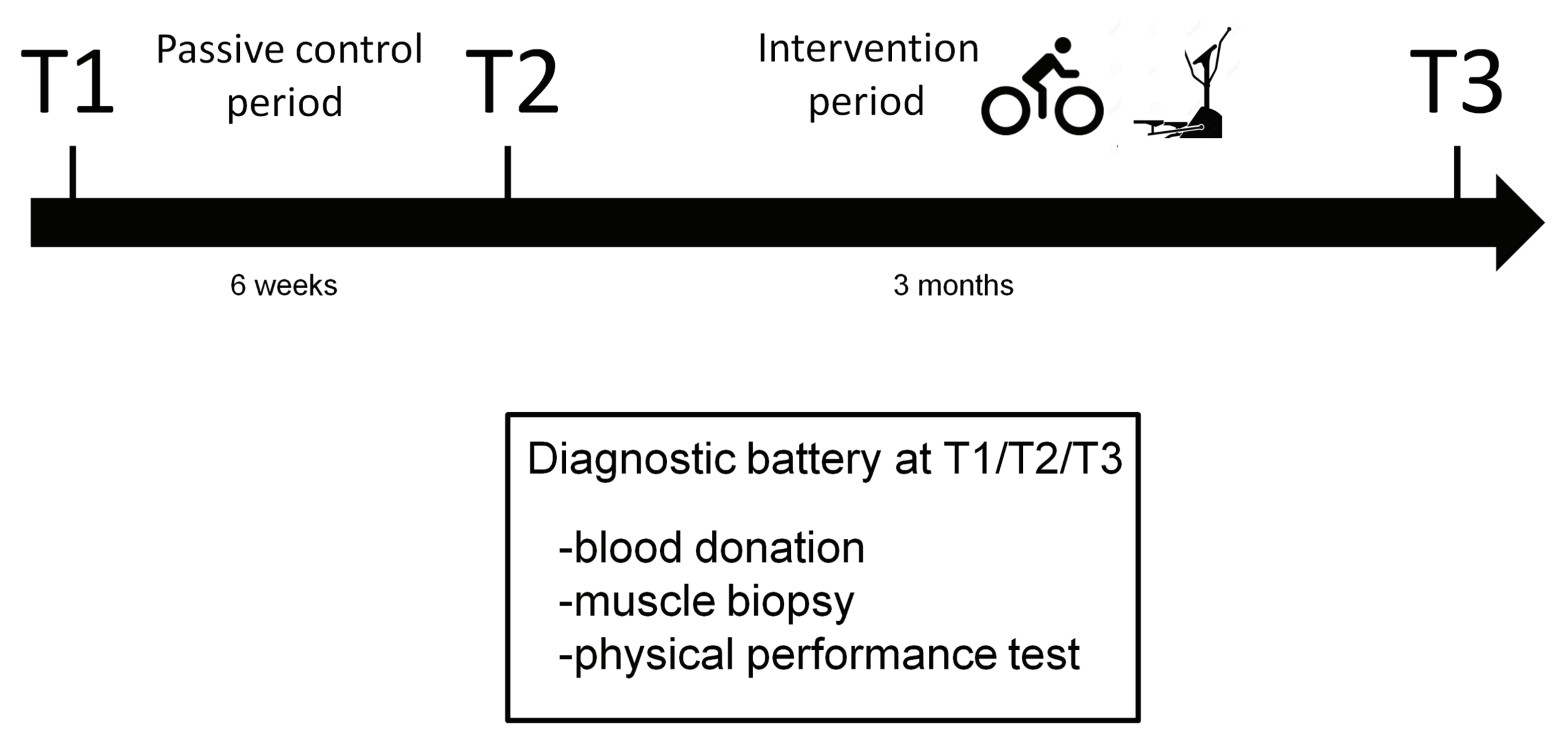
Study design.

Muscle biopsy samples from a previously conducted study were used for the miRNA analyses. The study has been approved by the Ethics Committee of the German Sport University Cologne. The study is registered in the German Clinical Trials Register (DRKS, clinical trial registration number: DRKS00022052). The protocol conformed to the provisions of the Declaration of Helsinki. Written informed consent was obtained from all subjects. The study has already been described in other papers of the working group (Brinkmann et al., 2017a,b, 2019). The study design is shown in Figure 1. Subjects underwent a medical check-up before participating in the study. A non-conventional study design was used for the study due to ethical reasons. The subjects’ data were analyzed 6 weeks pre-intervention (T1), 1 week pre-intervention (T2) and 3–4 days post-intervention (T3). The time period from T1 to T2 was used as a control period to detect possible time-dependent variations in the measured variables without the impact of physical training. In contrast to a conventional two-group design with a passive control group, this study design does not require the involvement of subjects who are inactive for the full duration of the study and who cannot improve their health by participating in the study. At T1, T2 and T3, venous blood was collected from the patients after a 12-h overnight fast and before medication intake in the early morning. Thereafter, a muscle sample was obtained from the M. vastus lateralis. A few days later, an endurance performance test was applied.

### Subjects

All patients were recruited *via* a newspaper advertisement. Seven overweight/obese [body mass index (BMI): 31.2 ± 2.1 kg/m^2^] and untrained (no regular exercise during the last 3 years) T2DM men (age: 61 ± 10 years) without insulin treatment participated in the study (Table 1). The duration of T2DM was 5 ± 7 years. All subjects were free of severe diabetic complications (e.g., nephropathy, neuropathy, and retinopathy) and/or any other severe cardiovascular diseases. Four patients had well-controlled arterial hypertension. Five of the patients took anti-diabetic drugs, four took anti-hypertensive drugs, two took anti-hyperlipidemic/anti-hypercholesterolemia drugs, and four took other drugs (anti-hypothyroidism-drugs, anti-allergic drugs, anti-platelet drugs).

**Table 1:** Patients’ fitness data and results from blood analysis.

Variable	T1 (6 weeks pre-training)	T2 (1 week pre-training)	T3 (post-training)	Overall significance: *p*-value	T1-T2: *p*-value	T2-T3: *p*-value	T1-T3: *p*-value
WHO test: maximal workload [W] (*n* = 6)	133 ± 34	129 ± 19	175 ± 27	**0.006**	1.000	0.018	0.042
VO_2peak_ [ml/min/kg] (*n* = 6)	23.99 ± 3.21	25.10 ± 2.78	29.32 ± 4.85	**0.042**	1.000	0.130	0.063
Plasma glycated hemoglobin (HbA1c) [%]	7.4 ± 1.8	7.45 ± 2.2	6.6 ± 0.3	0.158	/	/	/
Homeostatic model assessment for insulin resistance (HOMA-IR) index	7.3 ± 3.1	6.9 ± 4.2	6.6 ± 3.8	0.565	/	/	/
Body mass index [kg/m^2^]	31.17 ± 2.07	30.83 ± 2.21	30.94 ± 2.87	0.368	/	/	/
Serum fasting triglycerides [mg/dl]	198 ± 54	207 ± 49	200 ± 55	0.895	/	/	/
Serum fasting total cholesterol [mg/dl]	216 ± 47	224 ± 39	222 ± 40	0.156	/	/	/
Serum fasting low-density lipoprotein (LDL) [mg/dl]	151 ± 43	157 ± 32	133 ± 38	**0.018**	1.000	0.023	0.098
Serum fasting high-density lipoprotein (HDL) [mg/dl]	48 ± 8	50 ± 6	48 ± 5	0.565	/	/	/

Values are means ± SD. Bold values indicate significance.

### Training Intervention

Endurance training (cycling or exercising on an elliptical crosstrainer) was performed three times a week on non-consecutive days for 3 months. The training intensity was ~70–80% of the subject’s peak heart rate based on the endurance performance test conducted prior to the training period. The training was supervised by professional sports coaches. The time of the training bouts was gradually increased in the first 7 weeks from 20 to 50 min. In weeks 8–12, the duration was held constant. A 5-min warm-up and a 5-min cool-down were performed during each training session.

### Physical Performance Diagnostics

A symptom-limited cycling step test (WHO, 1968) was performed using the World Health Organization protocol (25 + 25 W every 2 min). In case of muscular exhaustion, angina pectoris, ischemia, paleness, cyanosis, arrhythmia, respiratory insufficiency, hypertension (systolic blood pressure > 250 mmHg or diastolic blood pressure > 115 mmHg), aberration, dizziness and/or coordination problems, the test was stopped. Spirometric measurements were carried out using the “ZAN 600 USB” system (nSpire Health, Longmont, Colorado, United States). The highest O_2_ consumption measured during exercise (60 s average value) was defined as peak oxygen uptake (VO_2peak_).

### Blood Analysis

Glucose, triglycerides, total cholesterol and lipoprotein levels were determined using the Cobas Mira analyzer (Hoffmann La Roche AG, Basel, Switzerland). Insulin and glycated hemoglobin (HbA1c) levels were determined in an external laboratory (Labor Dr. Wisplinghoff, Cologne, Germany). The homeostatic model assessment for insulin resistance (HOMA-IR) index was calculated as follows: (insulin [μU/ml] * glucose [mg/dl])/405.

### Muscle Biopsy Procedure

Skeletal muscle tissue was obtained from the M. vastus lateralis of the T2DM men by a needle biopsy (Evans et al., 1982). The muscle tissue sample was immediately placed in cryotubes, frozen in liquid nitrogen and stored at −80°C until further analysis.

### Selection of miRNAs

Micro-RNAs were selected through a literature search in PubMed. miRNAs that were found to be linked to insulin resistance/poor glycemic control (with evidence from *in vivo* or experimental *in vitro* cell model studies) and dysregulated in the skeletal muscle of T2DM patients (up-regulated or down-regulated) were selected for further analyses.

### miRNA Isolation and Quantification

miRNA isolation and profiling were performed similarly to the protocol used in the paper of Dahlmans et al. (2017). Total RNAs were extracted from muscle samples using the miRNeasy Mini kit (Qiagen, Hilden, Germany). cDNAs were then synthesized using the miRCURY LNA RT Kit (Qiagen). The premix of cDNAs, miRCURY LNA SYBR Green PCR Master Mix (Qiagen) and RNase-Free Water (Qiagen) was then added to predesigned 96-well Pick & Mix-miRCURY LNA miRNA Custom PCR Panels (Qiagen), according to the manufacturer’s guidelines. Finally, cDNAs were amplified using the “Stratagene Mx3005P” PCR system (Agilent Technologie, Santa Clara, California, United States). All samples were analyzed in duplicate. The x-fold change in the expression of a miRNA was calculated using the 2^−ΔΔct^ –method (Livak and Schmittgen, 2001). In repeated measurements, the values reflect changes from the baseline value (T1 values were set 1 = 100%). In correlation analyses, the values reflect the x-fold level of a miRNA in relation to the mean level of that miRNA in all subjects (the mean value was set 1 = 100%). Data of each sample were normalized to the mean of the expression values of miRNA-423-3p, SNORD48 and U6 snRNA.

### Sample Size

Because of the pilot nature of this study, a formal sample size calculation *a priori* based on the results of other studies was not performed. Biopsy samples from a former study have been used for analyses. Their number was limited. It is important to note that the participation rate in muscle biopsy studies is usually low due to possible experience of pain during the biopsy procedure and, in particular, when more than one biopsy is performed (as in the present study: three biopsies per person).

### Statistical Analyses

Data are presented as mean values ± standard deviations (SD). The “SPSS” program (v. 24.0, SPSS Incorporation, Chicago, Illinois, United States) was used for statistical analyses. Non-parametric (rank-based) hypotheses tests were performed throughout, as normality of continuous data distributions seemed to be questionable (skewness, outliers). The Friedman analysis of variance (ANOVA) was used for repeated measurements (T1-T2-T3). If found statistically significant, implemented post-hoc tests for pairwise comparisons were conducted (values of *p* were corrected for multiple testing with the Bonferroni correction method). In addition, Spearman’s correlation analysis was used to study the relationship between two variables. If found statistically significant, Spearman’s rank correlation coefficient rho was calculated. Significance was considered at *p* ≤ 0.05.

## Results

### Effects of the Training on the Patients’ Physical Fitness and Blood Variables

The T2DM patients’ endurance capacity (maximal workload during the WHO step test) improved significantly from pre- to post-training (T3 vs. T1 and T2; Table 1). The Friedman ANOVA also revealed an overall statistical significance for peak oxygen uptake (VO_2peak_), while post-hoc tests failed significance. One patient did not take part in the physical fitness diagnostics at T1 due to an acute infection. Glycemic control (glycated hemoglobin: HbA1c) and HOMA-IR index did not change significantly over time. However, it might be important to note that the HbA1c value was reduced in six of the seven patients at T3 vs. T2. Low-density lipoprotein (LDL) was reduced significantly from pre- to post-training (T3 vs. T2). However, post-hoc tests did not indicate significance for T3 vs. T1. There were no significant changes in BMI, triglycerides, total cholesterol or high-density lipoprotein (HDL) levels.

### Effects of the Training on the Patients’ miRNA Levels

**Figure 2:**
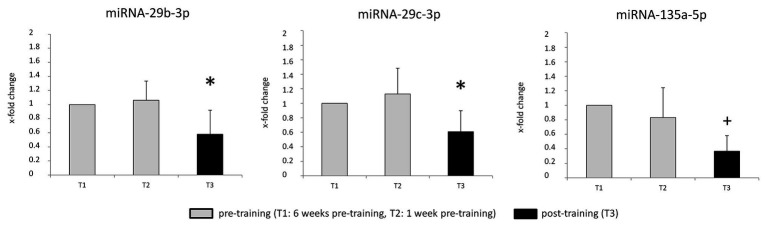
Skeletal muscle miRNA levels in the type 2 diabetes mellitus (T2DM) patients pre- and post-training. Values are means ± SD. ^*^Significantly different from T2 and T1. ^+^Significantly different from T1.

Friedman ANOVA and *post-hoc*-tests revealed that miRNA-29b-3p and miRNA-29c-3p levels were significantly reduced at T3 vs. T2 and T1 (miRNA-29b-3p: T3 vs. T2: −45%, T3 vs. T1: −42%; miRNA-29c-3p: T3 vs. T2: −46%, T3 vs. T1: −39%). The levels of miRNA-135a-5p were significantly reduced at T3 vs. T1 (−63%), but not vs. T2 (Table 2 and Figure 2). Levels of miRNAs-27a-3p, −29a-3p, −106b-5p, −143-3p, −144-3p, −194-5p, and −206 did not change as a result of the training.

**Table 2:** Skeletal muscle miRNA levels [relative expression of each miRNA: x-fold change to the baseline value (T1)] in the T2DM patients pre- and post-training.

miRNA	T1 (6 weeks pre-training)	T2 (1 week pre-training)	T3 (post-training)	Overall significance: *p*-value	T1-T2: *p*-value	T2-T3: *p*-value	T1-T3: *p*-value
miRNA-27a-3p	1.00 ± 0.00	1.39 ± 0.39	1.27 ± 1.11	0.156	/	/	/
miRNA-29a-3p	1.00 ± 0.00	1.11 ± 0.34	0.63 ± 0.33	0.066	/	/	/
miRNA-29b-3p	1.00 ± 0.00	1.06 ± 0.28	0.58 ± 0.34	**0.005**	1.000	0.023	0.010
miRNA-29c-3p	1.00 ± 0.00	1.13 ± 0.36	0.61 ± 0.29	**0.004**	1.000	0.004	0.048
miRNA-106b-5p	1.00 ± 0.00	2.49 ± 3.63	1.12 ± 1.50	0.066	/	/	/
miRNA-135a-5p (*n* = 5)	1.00 ± 0.00	0.83 ± 0.41	0.37 ± 0.22	**0.015**	1.000	0.173	0.013
miRNA-143-3p	1.00 ± 0.00	1.40 ± 0.85	3.55 ± 7.02	0.651	/	/	/
miRNA-144-3p	1.00 ± 0.00	5.91 ± 12.31	1.74 ± 3.27	0.156	/	/	/
miRNA-194-5p	1.00 ± 0.00	1.66 ± 1.53	1.15 ± 1.17	0.156	/	/	/
miRNA-206	1.00 ± 0.00	1.39 ± 0.63	1.21 ± 1.17	0.867	/	/	/

Values are means ± SD. Bold values indicate significance.

### Associations Between miRNA Levels, Glycemic Control/Insulin Sensitivity and Physical Fitness Among the Patients

**Figure 3:**
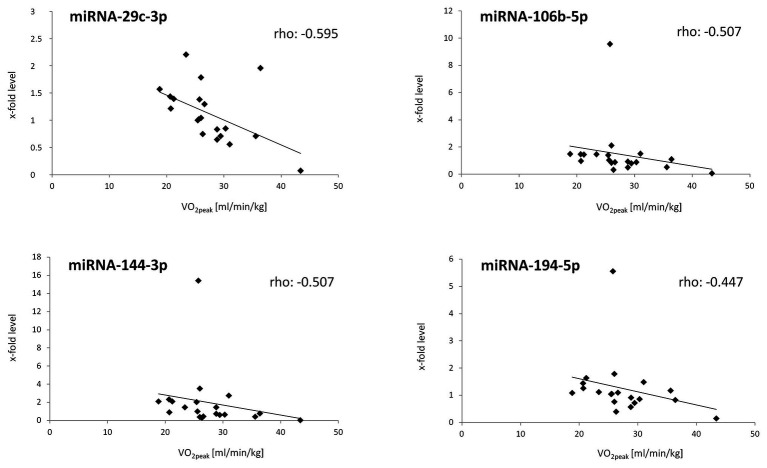
Significant rank-based correlations between miRNA levels and cardiovascular fitness (VO_2peak_). Spearmans’ correlation coefficients and trendlines were calculated.

Spearman’s rank correlation analyses were performed with all values from all time points (T1, T2, and T3 miRNA values with the respective corresponding HbA1c, HOMA-IR index or VO_2peak_ values). Analyses showed no significant associations between the miRNA levels and HbA1c values or HOMA-IR index values, while significant negative associations between the levels of miRNA-29c-3p, miRNA-106-5p, and miRNA-144-3p or of miRNA-194-5p and VO_2peak_ values (determined during the WHO step test) were found (Table 3 and Figure 3). Significant rank-based correlations were not observed for the levels of miRNAs-27a-3p, −29a-3p, −29b-3p, −135a-5p, −143-3p, or −206 with VO_2peak_ values.

**Table 3:** Rank correlations between miRNA levels and cardiovascular fitness in the T2DM patients.

miRNA	VO_2peak_	
	Significance: *p*-value	Correlation coefficient rho
miRNA-27a-3p	0.086	/
miRNA-29a-3p	0.062	/
miRNA-29b-3p	0.339	/
miRNA-29c-3p	**0.006**	−0.595
miRNA-106b-5p	**0.023**	−0.507
miRNA-135a-5p (*n* = 19)	0.576	/
miRNA-143-3p	0.930	/
miRNA-144-3p	**0.023**	−0.507
miRNA-194-5p	**0.048**	−0.447
miRNA-206	0.454	/

Bold values indicate significance.

## Discussion

This study analyzed the influence of a physical training intervention on the levels of some skeletal muscle miRNAs in T2DM patients, as miRNAs have been discussed as new important regulators of glycemic control (Jones et al., 2017; Zhang et al., 2019). The study provides evidence that regular physical (endurance) training is beneficial for the regulation of skeletal muscle miRNAs that have been demonstrated to negatively affect glucose disposal. We found that miRNA-29b-3p, miRNA-29c-3p and miRNA-135a-5p levels are significantly down-regulated in the skeletal muscle of T2DM patients through training. Furthermore, correlation analyses reinforce the assumption that improving one’s physical fitness could contribute to beneficial regulation of some of the analyzed miRNAs. There were significant negative associations between miRNA-29c-3p, −106b-5p, −144-3p and −194-5p levels and cardiovascular fitness. The fact that three out of the four miRNAs (miRNA-106b-5p, −144-3p, and −194-5p) that correlated with cardiovascular fitness were not significantly altered by the training could be explained by the fact that the dose of training in the present study was probably not sufficient for changing their levels. By contrast, some miRNA levels (miRNA-29b-3p and miRNA-135a-5p) were altered by the training, but did not correlate with the subjects’ cardiovascular fitness, which could be explained by the fact that there can be a high inter-individual variability in some miRNA levels at roughly the same fitness level.

Skeletal muscle is the pre-dominant site of prostprandial insulin-mediated glucose uptake and is therefore of high metabolic importance (DeFronzo and Tripathy, 2009). It has been discovered that miRNAs are important for the glucose metabolism in several tissues, among others in skeletal muscle (Massart et al., 2016). miRNAs are small RNA molecules (containing about 18–24 nucleotides) and can regulate gene expression post-transcriptionally by pairing with messenger RNAs, resulting in silencing of these messenger RNAs (Yu et al., 2016). miRNAs can be transported in vesicles from one organ to another and can thus be involved in organ cross-talks (Guay and Regazzi, 2017; Boilard, 2018).

There is evidence to suggest that some miRNAs can negatively affect glucose metabolism in skeletal muscle. miRNAs -27a-3p, −29a-3p, −29b-3p, −29c-3p, −106b-5p, −135a-5p, −143-3p, −144-3p, −194-5p, and −206 were all found to be negatively linked to variables of glycemic control (Gallagher et al., 2010; Jordan et al., 2011; Karolina et al., 2011; Zhang et al., 2013, 2017; Latouche et al., 2016; Zhou et al., 2016; Dahlmans et al., 2017).

Regarding the miRNAs that were altered by regular physical training in the present study, Dahlmans et al. (2017) have shown a negative correlation between skeletal muscle miRNA-29b/miRNA-29c and peripheral insulin sensitivity in human study participants (involving T2DM patients, non-diabetic obese and lean subjects, athletes). Furthermore, Dooley et al. (2016) demonstrated that miRNA-29c-deficient mice were protected against the onset of diet-induced insulin resistance. Agarwal et al. (2013) revealed that miRNA-135a can reduce insulin receptor substrate (IRS)-2 levels in C2C2 cells and that *in vivo* silencing of miRNA-135a improves glucose tolerance and alleviates hyperglycemia in mice.

Although an association between the miRNAs that were analyzed in the present study and glucose metabolism can be assumed, no significant correlations between the above-mentioned miRNA levels and glycemic control (HbA1c) or insulin resistance (HOMA-IR index) were found. This may be due to the fact that only a relatively small range of HbA1c and HOMA-IR index values was available. It can be speculated that correlations become statistically significant if there is a much wider range of values and if both healthy individuals and patients with insulin resistance are included in the analyses.

Numerous studies have found that the above-mentioned miRNAs can all be dysregulated in the skeletal muscle of T2DM patients showing an up-regulation or down-regulation compared with the levels of non-diabetic control subjects (Gallagher et al., 2010; Jordan et al., 2011; Agarwal et al., 2013; Latouche et al., 2016; Dahlmans et al., 2017). However, the study results are inconsistent, sometimes also indicating no significant difference (Dahlmans et al., 2017). These inconsistent findings warrant further investigation. To what extent the duration of the disease or the presence of diabetic complications could influence miRNA regulation in T2DM patients should be clarified. Regular physical activity could also influence the miRNA levels in T2DM patients. Studies involving healthy subjects revealed changes in circulating miRNA levels, as well as in miRNA levels in skeletal muscle (Domańska-Senderowska et al., 2019).

Changes in the miRNA profile of T2DM patients could be co-responsible for improvements in insulin sensitivity and glycemic control that are usually evident following physical training (Umpierre et al., 2011; Way et al., 2016). We have shown that in six of the seven patients the HbA1c value was reduced as a result of the performed endurance training. However, glycemic control (HbA1c) as well as insulin sensitivity (HOMA-IR index) were not changed significantly. In future studies, it would be useful to also perform the oral glucose tolerance test or to use the hyperinsulinemic-euglycemic clamp technique to determine the patients’ insulin sensitivity to further substantiate the present findings and possible relationships between changes in miRNA levels and changes in glycemic variables.

A clear limitation of the study is the relatively low number of patients included and the results should be interpreted with caution. However, this pilot study gives first insights into the training-induced regulation of skeletal muscle miRNAs in diabetes patients, and the present results could be helpful for the sample size calculation for future large-scale studies. The subjects` medication intake could have influenced the present results. Among the patient group, five men took anti- diabetic drugs. It has been shown that metformin can affect the miRNA profile in T2DM patients (Zhou et al., 2015; Demirsoy et al., 2018). Finally, it cannot be excluded that other types of training (strength training or combined endurance/strength training) or other training intensities or durations would have influenced the results differently.

## Conclusion

This preliminary study indicates that regular physical exercise and improving one’s physical fitness could be beneficial for the regulation of skeletal muscle miRNAs in patients with T2DM. Future studies are needed to clarify whether or not changes in the miRNA profile can affect glycemic control in T2DM patients.

## Data Availability Statement

The raw data supporting the conclusions of this article will be made available by the authors, without undue reservation.

## Ethics Statement

The study involving human participants was reviewed and approved by the Ethics Committee of the German Sport University Cologne. The patients/participants provided their written informed consent to participate in the study.

## Author Contributions

CB designed the study. SS performed the literature search and analyses and wrote the manuscript. WB and H-GP contributed reagents, materials, and analysis tools. BS-K and TS performed medical examinations. WB, KB, and CB reviewed and edited the article. All authors contributed to the article and approved the submitted version.

### Conflict of Interest

The authors declare that the research was conducted in the absence of any commercial or financial relationships that could be construed as a potential conflict of interest.
